# The theory of orchid and dandelion offers a new subtyping framework for cognitive aging

**DOI:** 10.18632/aging.204955

**Published:** 2023-07-28

**Authors:** Sylvain Moreno, Emma Rodrigues, Faranak Farzan

**Affiliations:** 1 School of Interactive Arts and Technology, Simon Fraser University, Surrey, BC, Canada

**Keywords:** subtyping, trajectories, healthy cognition, cognitive aging, heterogeneity

The last 30 years of cognitive aging research have focused on finding what normal cognitive aging is. What does it mean to age at the cognitive level? Prominent researchers such as Salthouse have argued “that normal cognitive aging is characterized by nearly linear declines from early adulthood” [1], defining the healthy cognitive aging population as one group. Other scientists, such as Buckner and Lindenberger, dispute this fact and describe cognitive aging as highly variable, presenting discoveries related to brain functioning and plasticity [2, 3]. However, no matter what framework is advanced, studies in cognitive aging treat participants as homogeneous (i.e., younger vs. older adults experimental design). We observe a “one size fits all” approach that dominates the literature. Such an approach is not specific enough, potentially causing confusion and advancing potentially contradicting findings [4]. In the context of human research, Molenaar demonstrated that the factors that best describe a group would not be depictive of an individual of the same group [5]. The latter implies that intrinsic data heterogeneity is ignored or treated as noise. As a solution, several scientists have proposed a different approach to cognitive aging, which breaks away from the scientific paradigms used before and looks at cognitive aging in terms of population subtyping. This new conceptualization requires a different methodological approach in which cognitive aging is not linear and is studied in its multiplicity instead of only its variability. Subtyping can be defined as categorizing a group of participants based on characteristics issued from their genotype and/or phenotype. Cognitive aging subtyping aims to understand better the heterogeneity of cognitive abilities that people experience as they age.

Previous research has been limited in implementing such a framework due to methodological and technical difficulties resulting in the use of linear analysis models and the collection of low number of participants. With new initiatives in recording highly heterogeneous data in a large pool of participants such as UK Biobank (https://www.ukbiobank.ac.uk/), or Canadian Longitudinal Studies Aging (https://www.clsa-elcv.ca/) coupled with the recent progress in implementing new analytic techniques, we have a unique opportunity to implement this new scientific paradigm. Taking advantage of those large-scale data collection initiatives, researchers can investigate the possibility of heterogeneity in cognitive aging, not as ‘noise’ as it was considered in the past, but as a central attribute of our measurements. Several research groups have recently reported findings describing the significant heterogeneity in cognitive aging. For example, Gorbach et al. and Nyberg et al. reported considerable heterogeneity at the structural level of the aging brain [6, 7]. From those results, different subtyping paradigms have started to be proposed. Nyberg et al. showed that the aging population could be more accurately described as three groups depending on the structural brain area atrophy rate [7]. Also, Wu et al. investigated associations between cognitive aging trajectories, sociodemographic characteristics, and dementia [8]. Their findings identified four to seven subtypes depending on the selected cognitive domain. Through those examples, we understand that the question is not about whether aging subtypes are necessary but rather how to identify subtypes. We recently proposed a novel method of subtyping in healthy cognitive aging [9]. Using the Health Retirement Study (https://hrs.isr.umich.edu/about), we explored the potential effects of lifestyle activities (i.e., environment) on individuals’ subtypes of cognitive aging. Using a stratification method based on cognitive scores, our results showed that lifestyle factors like smoking or drinking did not impact older adults with cognitive scores close to the group average. By comparison, older adults with high or low cognitive scores were highly impacted by lifestyle factors. This study revealed a surprising result: the environment did not impact a subtype of older adults. Similar results were found in research on cognitive development. Indeed, Boyce and Ellis advanced a theory based on differential susceptibility that accounts for biological sensitivities in childhood to various harmful and protective environmental effects and their impact on development into adulthood [10]. They proposed a developmental dichotomy or subtyping to describe their pediatric patients: the theory of *orchids* and *dandelions*. Boyce and Ellis use a flower metaphor to express individual environmental susceptibility. Orchid individuals are more sensitive to the environment: they flourish under favourable environmental conditions but are more affected by adverse environmental conditions. Conversely, dandelion individuals are less sensitive to their environment: they do not flourish to the same degree as orchids in optimal conditions but are more resilient to poor environmental conditions. Rodrigues et al. further demonstrated the translational utility of subtyping by extending the orchid and dandelion theory to the cognitive aging population and providing a new framework to understand cognitive aging [9].

The empirical evidence for investigating heterogeneity in cognitive aging has been growing, and the implementation of population subtyping is becoming a promising avenue for future research. The theory of *orchids* and *dandelions* is a potential framework for researchers to adopt. With the emergence of extensive longitudinal database studies, we can better study and understand the different cognitive aging profiles. More research is needed to understand the complex dynamics of cognitive aging fully, but it is clear that the “one size fits all” model or a linear approach no longer applies.
